# Clinician’s attitude towards clozapine prescription

**DOI:** 10.1192/j.eurpsy.2023.2152

**Published:** 2023-07-19

**Authors:** O. Brugue, M. Gonzalez, L. Moreno, C. Ivorra, R. Hernandez, S. Cepedello, J. Labad

**Affiliations:** Psychiatry, Hospital de Mataró, Mataró, Spain

## Abstract

**Introduction:**

Current clinical guidelines recommend the use of clozapine for the treatment of refractory schizophrenia, present in up to a third of patients with this disease. Despite the evidence, the data point to low prescription, underdosing, and delayed initiation.

**Objectives:**

The objective of the study is to elucidate which factors may interfere in clozpine prescription.

**Methods:**

This is a cross-sectional observational study, carried out using a survey designed specifically for it.

It was answered online by seventy psychiatrists affiliated with the Catalan Society of Psychiatry.

**Results:**

More than half admitted having prescribed two or more antipsychotics without having previously ruled out pseudorefractoriness through depot treatment. 70% recognized the need for monitoring as the main prescription barrier, while the main reason for withdrawal was its adverse effects. The most alarming was considered agranulocytosis, with drooling, drowsiness and weight gain being the most reported.

Statistically significant differences (p=0.031) were found in relation to the years of experience and the device where clozapine was preferred to be started: <10 years in hospital, 10-20 years in partial hospitalization and >20 years outpatient office.

Statistically significant differences were observed in the preference of the device for its initiation depending on the usual work device: hospitalization (p<0.000) and partial hospitalization (p=0.046) preferred to schedule it from their respective devices, without any preference in consultations.

The level of experience and the most reported side effect were statistically significant: for the newest psychiatrists it was weight gain (p=0.031), without presenting differences in the rest of the groups.

**Conclusions:**

Clozapine is the psychoactive drug of choice in refractory schizophrenia, so efforts should be devoted to reducing prescription barriers, offering training on its management and innovating forms of monitoring to promote its use.

**Disclosure of Interest:**

None Declared

